# A Toolkit for bulk PCR-based marker design from next-generation sequence data: application for development of a framework linkage map in bulb onion (*Allium cepa* L.)

**DOI:** 10.1186/1471-2164-13-637

**Published:** 2012-11-19

**Authors:** Samantha Baldwin, Roopashree Revanna, Susan Thomson, Meeghan Pither-Joyce, Kathryn Wright, Ross Crowhurst, Mark Fiers, Leshi Chen, Richard Macknight, John A McCallum

**Affiliations:** 1 The New Zealand Institute for Plant & Food Research Limited, Private Bag 4704, Christchurch, New Zealand; 2 Department of Applied Computing, Faculty of Environment, Society and Design, Lincoln University, PO Box 84, Lincoln, 7647, New Zealand; 3 Biochemistry Department, University of Otago, P.O. Box 56, Dunedin, 9054, New Zealand

**Keywords:** Marker, Onion, Genetic mapping, Next generation sequencing, SNP

## Abstract

**Background:**

Although modern sequencing technologies permit the ready detection of numerous DNA sequence variants in any organisms, converting such information to PCR-based genetic markers is hampered by a lack of simple, scalable tools. Onion is an example of an under-researched crop with a complex, heterozygous genome where genome-based research has previously been hindered by limited sequence resources and genetic markers.

**Results:**

We report the development of generic tools for large-scale web-based PCR-based marker design in the Galaxy bioinformatics framework, and their application for development of next-generation genetics resources in a wide cross of bulb onion (*Allium cepa* L.). Transcriptome sequence resources were developed for the homozygous doubled-haploid bulb onion line ‘CUDH2150’ and the genetically distant Indian landrace ‘Nasik Red’, using 454™ sequencing of normalised cDNA libraries of leaf and shoot. Read mapping of ‘Nasik Red’ reads onto ‘CUDH2150’ assemblies revealed 16836 indel and SNP polymorphisms that were mined for portable PCR-based marker development. Tools for detection of restriction polymorphisms and primer set design were developed in BioPython and adapted for use in the Galaxy workflow environment, enabling large-scale and targeted assay design. Using PCR-based markers designed with these tools, a framework genetic linkage map of over 800cM spanning all chromosomes was developed in a subset of 93 F_2_ progeny from a very large F_2_ family developed from the ‘Nasik Red’ x ‘CUDH2150’ inter-cross. The utility of tools and genetic resources developed was tested by designing markers to transcription factor-like polymorphic sequences. Bin mapping these markers using a subset of 10 progeny confirmed the ability to place markers within 10 cM bins, enabling increased efficiency in marker assignment and targeted map refinement. The major genetic loci conditioning red bulb colour (*R*) and fructan content (*Frc*) were located on this map by QTL analysis.

**Conclusions:**

The generic tools developed for the Galaxy environment enable rapid development of sets of PCR assays targeting sequence variants identified from Illumina and 454 sequence data. They enable non-specialist users to validate and exploit large volumes of next-generation sequence data using basic equipment.

## Background

### Marker design from genome variants

Economical third generation sequencing technologies now permit the deep sampling of variation from poorly characterized species, providing a wealth of data to enable genetic studies [1]. In practice, a dearth of accessible, scalable and biologist-friendly bioinformatics tools for exploiting these large-scale data restricts application of these sequencing technologies in minor species and institutions lacking bioinformatics infrastructure [2].

The typical goal in sampling sequence variation is to detect variants for diagnostic and/or functional studies, most commonly single-nucleotide polymorphisms (SNPs) and insertion-deletion polymorphisms (indels). Although a myriad of technologies have been developed for interrogation of SNPs, the most widely accessible technologies are those based on PCR. Among the most simple and robust means to interrogate SNP variation is that of cleaved amplified polymorphic sequences (CAPS; also known as PCR-RFLP, snipSNPs), where sequence variants are revealed by post-PCR cleavage of amplicons with restriction enzymes [3]. Addition of engineered mismatches in primer sequences can allow detection of SNPs that do not condition restriction polymorphisms [4]. High-resolution melting (HRM) of small amplicons in the presence of intercalating dyes is increasingly used as a means to reveal sequence variation [5,6], and has the advantage of being a closed-tube assay. Although both approaches could be used to validate and evaluate polymorphism at variant sites identified by deep sequencing, the principal barrier to achieving this on a useful scale is design of flanking PCR primer pairs for large numbers of targets.

Web-based tools which have been reported for design of CAPS markers from small numbers of sequences include BlastDigester [7], SNP2CAPS [8] and SNP Cutter [9]. None of these tools readily scale to large volumes of NGS data. Bulk design of primer sets to SNPs or other genome targets can be performed using the Primer3 executable [10]. Although the use of this command-line tool can be simplified by use of programming interfaces such as those provided by BioPerl [11] or BioPython [12], such scripting is usually performed by specialists and is generally reported in the scientific literature as ‘custom scripts’ without code or detail sufficient to permit reproducibility by non-specialists. Web-based tools suitable for larger-scale primer design include SNP-RFLPing [13] for mammalian SNP assay design and PRIMEGENS-w3 [14] which provides a variety of options for assay and probe design, especially for well-characterized genomes. Neither of these tools provides source code and the web-based tools they provide are constrained to specific uses in well-characterized genomes. The principal challenge faced by developers of such traditional web applications has been to support the diversity of input data and possible applications by biologists.

Web-based bioinformatics workflow frameworks, such as GenePattern [15] and Galaxy [16-18], now provide a means to share biologist-friendly tools and complex workflows for bioinformatics tasks such as PCR-based primer design. Importantly, they encourage a modular approach to code and tool development, providing greater flexibility to accommodate diverse inputs and goals. These features support reproducibility of bioinformatics methods by specialist tool developers and non-specialist end-user scientists. We previously reported the potential for enabling PCR-based primer design in web-based bioinformatics frameworks when we adapted MISA scripts [19] for simple sequence repeat (SSR) marker design to Galaxy [20].

### Onion genome resources

Although onion and shallot (*Allium cepa* L.) are among the most widely cultivated and traded vegetable crops, knowledge of their genomes, population structure and genetic architecture of key traits is limited [21]. There is strong need for applied genomic resources to enable quality control of hybrid seed, inform genetic resource mining and to accelerate genetic analysis and improvement of consumer and sustainability traits. Laboratories engaged in onion research and breeding typically have limited technical and financial resources, as is common in those researching second-tier crops and non-model species. Therefore it is desirable that marker assays can be implemented in laboratories with basic equipment.

The genomes of onion and related Allium crops such as garlic (*Allium sativum*) and bunching onion (*Allium fistulosum*), are very large (10–20 Gbp) and even transcriptome sequencing has been limited to modest EST projects [22,23]. A partial (0.3N) onion BAC library [24] provided insights into gene structure and genome composition, most notably the very low gene density of one gene per 168 kb [25]. The initial genetic linkage map ‘BYG15-23 x AC43’ developed by Havey and colleagues using RFLP markers remains as the key reference map [26-28]. Notably, this map revealed a very high level of dominant RFLP, suggesting that the large genome size of onion is associated with high levels of gene duplication. Genetic stocks used to date for development of onion mapping populations have generally been inbred lines that have typically been only subjected to one generation of self-pollination. The high levels of residual heterozygosity have previously greatly complicated marker development and sequence analysis in onion. Although a number of researchers have produced doubled haploid onion (DH) lines, these have in general suffered from poor seed set [29]. The development of highly fecund DH lines from long-day US onion varieties by Alan et al. [30,31] now provides an opportunity to use homozygous, distributable reference lines for onion genetics and genomics.

In the present study we sought to develop PCR-based genetic markers that were easily transferable among the *Allium* research community, based on transcriptome sequence polymorphisms segregating in a wide bulb onion cross. Inspection of the data revealed potential for large-scale development of robust, low-technology PCR-based markers, which was enabled by a set of simple bioinformatics tools usable in the Galaxy workflow environment. We used these markers to develop a framework map spanning much of the genome. We further tested the utility of these by conducting targeted design and bin mapping of transcription factor candidates [32]. The genomics resources developed in this study provide a framework for genetic analysis and genome sequencing in onion. The bioinformatics tools are applicable for any biologist requiring large-scale PCR-based variant validation and assay design from modern sequencing platforms.

## Results and discussion

### cDNA sequencing and variant discovery

We set out to discover SNPs which could be used to develop genetic markers revealing allelic variation between the genetically distant onion parent lines used to develop a large F_2_ mapping population. To maximise the amount of novel sequence obtained using GS-FLX sequencing, we normalized the cDNA samples to reduce the most abundant transcripts. BLASTX analysis of pilot 1/16 plate GS-FLX sequencing runs of normalised shoot cDNA samples from ‘CUDH2150’ (SRX031644) and ‘Nasik Red’ (SRR073449) revealed that high-abundance transcripts (RuBISCO, histones, photosystem components and ribosomal sequences) comprised 1.8% and 2.2% of reads respectively, indicating acceptable normalisation. A full plate of GS-Titanium sequence was generated from the homozygous line ‘CUDH2150’ (899438 reads with a modal length of 400 bp; SRX031645) to provide a working reference assembly and a plate of GS-FLX sequence was obtained for ‘Nasik Red’ (578117 reads, modal length 255 bp; SRR073447) for variant discovery. We chose 454 sequencing chemistry for greater read length, since the genome of onion has not been sequenced and there is only limited transcriptome data [22,23]. This reference assembly of ‘CUDH150’ contains 24106 contigs with N50 contig size of 677 bp representing 85% of the total reads. Mapping ‘Nasik Red’ reads onto these contigs revealed 14467 and 2369 indels between the parental lines, representing one variant per 740 bp. Due to the high degree of duplication in onion, estimates of SNP frequency based on this assembly should be interpreted conservatively.

### Bioinformatics and marker design

Preliminary inspection of variant data revealed numerous SNPs conditioning restriction polymorphisms suitable for CAPS marker design. However, a literature survey failed to reveal any published code or tools that could be readily used to facilitate identification of these and to design flanking PCR primer sets on large data sets. Therefore, prototype scripts were developed using BioPython [12] to identify SNPs conditioning restriction polymorphisms for enzymes known to perform well in PCR buffers, based on our prior experience developing CAPS markers in onion [33]. Mining of the variant data revealed a total of 2395 polymorphic restriction sites, the most abundant being those revealed by TaqI (438), AluI (401), RsaI (381), DpnII (321), HinfI (281) and HaeIII (147). Using the BioPython interfaces to EMBOSS and Primer3, custom scripts were used to design flanking primer pairs to variant features with masking of non-target variant sites.

Based on experience gained in developing tools for SSR marker design [20], we modified the prototype scripts to enable more general usage in the Galaxy workflow environment [34]. The detection of CAPS polymorphisms and design of primers was separated into two tools, which were modified to use Galaxy interval format and GFF3/GVF formats [35] as the input and output formats. Helper scripts were developed to enable conversion of VCF [36] and Roche gsMapper 454HCDiffs.txt variant formats to GFF3 formats. CAPS detection and primer design tools were modified to use iterators to provide efficient memory usage with genome-scale data. Additional tools were developed to parse EMBOSS primersearch output for conducting electronic PCR and PATMAN [37] for mapping primers back to sequences. The tools are available for installation to any Galaxy installation at Galaxy Toolshed (http://toolshed.g2.bx.psu.edu) as repository ‘pcr_markers’ (http://toolshed.g2.bx.psu.edu/repos/john-mccallum/pcr_markers/). The scripts may also be obtained from GitHub (https://github.com/cfljam/galaxy-pcr-markers) for direct use from the command-line.

Workflows for using these tools to design CAPS markers from Illumina (vcf files) or Roche 454 data (gsMapper 454HCDiffs.txt files) are provided in the Galaxy Toolshed repository and are described in on-line documentation at GitHub (https://github.com/cfljam/galaxy-pcr-markers/wiki). Polymorphism information from Illumina (samtools vcf) or Roche 454 (454HCDiffs.txt) read mappers is converted to gff3 format using vcf2gff or gsmapper2gff tools. The resulting gff3 and reference fasta file are provided as input to the CAPS detection tool. The list of desired features can then be filtered out and cut from this tabular output file using standard Galaxy tools and provided to the primer design tool. This provides tabular output containing primer pairs. These or existing primer pairs may be tested for redundancy and specificity by electronic PCR against reference sequences using EMBOSS primersearch or individual primers mapped onto sequences using PATMAN.

### Construction of a genetic linkage Map

A total of 376 primer sets were designed to target putative restriction polymorphism, indel or SNP variants. Of these, 91% of the sets amplified products under standard conditions with no optimisation (Table 1). HRM markers exhibited the most polymorphism during screening but were frequently unsuitable for mapping in the F_2_ population because of difficulty in reliably resolving homozygotes. By contrast, the CAPS and indel markers were robust and reproducible. These markers are the most transferrable as they only require standard PCR and gel analysis equipment available in all genetics laboratories. Overall, 58% of the markers designed from EST sequence which amplified in genomic DNA were polymorphic between the parent lines. This SNP conversion rate is higher than the 25% found in onion previously [22] and similar to the 51% found for pine [38]. Koepke et al. [39] reported a validation rate of 30.5% from HRM primers designed using 3^′^ UTR sequencing data.

**Table 1:** Summary of marker validation outcomes for the indel and SNP markers (CAPS and HRM) generated in this study

**Marker**	**Number assessed**	**Amplified**	**Multi-locus (%)**	**Polymorphic between Nasik Red and CUDH2150 (%)**	**Mapped in F**_**2**_
Indel	22	21 (95%)	0	11 (50%)	8 (36%)
CAPS	167	144 (86%)	32 (19%)	90 (54%)	57 (34%)
HRM	187	172 (92%)	33 (18%)	104 (56%)	16 (9%)
Total	376	337 (90%)	65(19%)	195(58%)	

Despite the lower success rate of HRM markers, these are an appealing marker class for design and screening in bulk using these approaches. Importantly, for studies of the large and duplicated onion genome, the use of a homozygous DH during screening permits ready confirmation that the amplicon derives from a single locus and heterozygosity is easily discerned in F_1_ or population samples. Implementing melt prediction methods such MELTSIM [40] and unlabeled probe design are two obvious strategies that could be used to improve outcomes for bulk HRM marker design in Galaxy.

**Figure 1:**
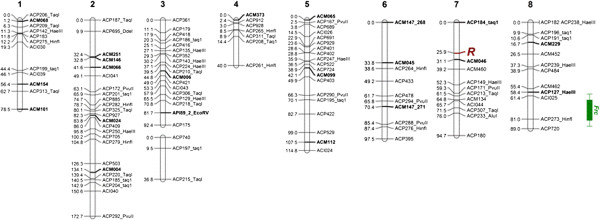
**Linkage map of ‘Nasik Red’ x ‘CUDH2150’ F**_**2**_**onion population.** Molecular marker classes include CAPS (ACP prefix, interrogating enzyme follows underscore), HRM (ACP prefix only), indels (ACI prefix) and microsatellites (ACM prefix). The QTL for *Frc* (bars denote 1 and 2 LOD confidence intervals) and the morphological colour locus *R* are also shown.

Of the 376 markers tested, 93 were assigned to 1 of 9 linkage groups assigned to the 8 chromosomes of *A. cepa* anchored using previously published markers (Figure 1; Additional file 1: Table S1). The overall map length was 808 cM. The map and underlying data can also be accessed at alliumgenetics.org [41]. The markers appeared to show both some overlap at particular positions, which may indicate redundancy or gene clusters but were also spread across the genome with an average spacing between markers of 7.5 cM. The major genetic loci conditioning red bulb colour (*R*) and fructan content (*Frc*) were located on this map by QTL analysis on chromosomes 7 and 8 respectively, as expected [42-44]. This resource is useful across onion germplasm since the anchor markers used here have been tested in other mapping populations, allowing the linkage maps to be aligned for comparative mapping using the CMap tool [45] provided at http://alliumgenetics.org[41]. The map was then used as a reference to select a subset of genotypes for bin mapping [46,47] to facilitate rapid marker screening and targeted map development. A set of 10 genotypes was identified for selective genotyping (bin mapping) using MapPop [32], providing an approximate bin length resolution of 8.8 cM.

### Design and Bin mapping of transcription factor markers

**Figure 2:**
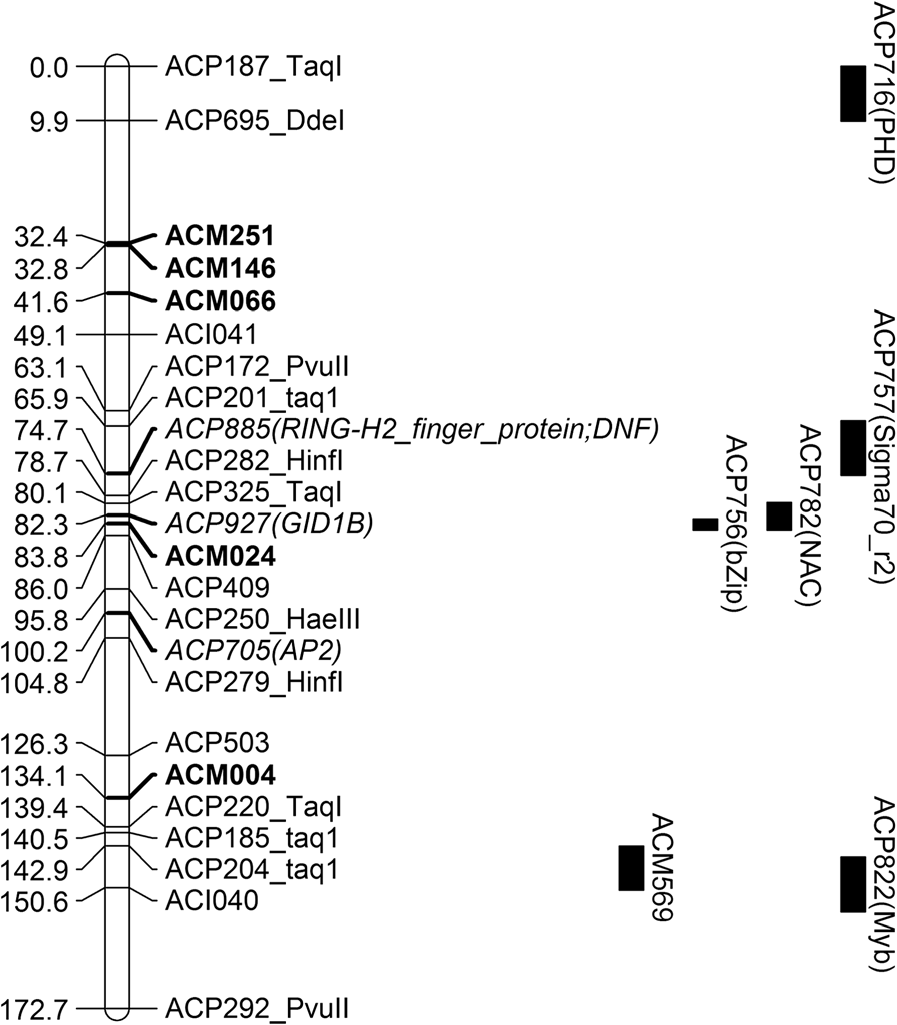
Genetic linkage map of chromosome 2 for ‘Nasik Red’ × ‘CUDH2150’ showing the assigned locations of bin mapped markers targeting transcription factor-like sequences.

Using a Galaxy workflow, SNPs were identified by mapping ‘Nasik Red’ reads to ‘CUDH2150’ contigs that showed significant matches to transcription factor motifs. Transcription factors have been implicated in regulating genes in pathways controlling many key economic traits in crops including stress response, flowering and colour. A total of 95 primer sets (27 HRM markers and 68 CAPS markers) were designed to flank these polymorphisms. Of these, 84 amplified products, including 13 that amplified multiple loci. Of the remaining, 31/71 (44%) exhibited polymorphism between parent lines and could be assigned to the genetic map by selective or complete genotyping in the mapping population (Figure 2; Additional file 1: Table S1 and Additional file 2: Table S2). This confirmed the utility of these tools for targeted design of markers to sets of candidate gene variants. The genetic resources developed also allowed the markers to be efficiently mapped to the onion genetic map to within 10 cM using just 10 lines from the population. The combination of bulk marker design and bin mapping now allows a more targeted approach to onion genetic map improvement through saturating regions of interest or low coverage.

## Conclusions

The tools for PCR-based assay design we present provide a ‘missing link’ to facilitate access to the wealth of sequence variant data from modern sequencing technologies by researchers with limited informatics and laboratory equipment. Importantly they are provided with source code and customised for use in a web-based framework to permit community improvement and use by non-specialists. The ability to easily develop custom panels of SNP markers for interrogating genes or genome regions of interest will complement modern genetic strategies that identify candidate variants through deep sequencing of population samples.

The success and practical utility of our assay design highlights the benefits of implementing bioinformatics applications in a reproducible research environment. Prior studies have either not disclosed code or methods in a reproducible form, or have exposed tools through web interfaces specialised for particular application domains. In contrast to the previous web-based solutions for CAPS design that enable a multistep pipeline, implementation in Galaxy requires tools for individual steps and encourages sharing of these through public repositories. This provides greater flexibility for researchers with diverse technologies and needs.

The practicability of CAPS markers in onion supports previous reports in *Arabidopsis*[3], *Caenorhabditis*[48,49] and human [13,50]. Now that such polymorphisms may be readily designed using NGS data these may become an appealing marker class for other non-model organisms.

The strategy employed in this study integrating a DH reference parent line, NGS variant data and bulk marker design is a next-generation strategy for onion genetics that has proven much faster, cheaper and less technically demanding than marker development in previous studies of onion [28,51,52]. Because the sequence resources, Galaxy tools and parental lines forming the basis of this work are publically available, they will provide a reproducible framework for future onion genome research. The very large family sizes, high levels of polymorphism and segregation for multiple traits make these families ideally suited for field-based population studies and fine mapping. We are currently using the framework map for genetic analysis of metabolic and developmental traits, and see potential for supporting genome sequencing of onion. Reduced representation sequencing of the gene space of a DH line such as ‘CUDH2150’ using Cot-based methods [53,54] or methyl filtration [55] is likely to be the first phase of developing an onion genome reference sequence. These new mapping and marker development resources will support the bin mapping and fine mapping strategies required to align contigs with the genetic and physical maps of *Allium*.

## Methods

### Plant materials and propagation

The doubled haploid onion line ‘CUDH2150’ was provided by Cornell University [30,31] and the heterozygous landrace ‘Nasik Red’ (PI271311) was obtained from the USDA ARS Plant Genetics Resources Unit (Cornell University, Geneva, NY). Two individual flowering plants were cross-pollinated by blowflies and multiple F_1_ plants were individually self-pollinated to generate F_2_ families. Two F_1_ plants spontaneously produced topset bulbils, which were replanted and mass-pollinated to provide two very large F_2_ families. Samples of these families were grown at Lat 42 deg S near Christchurch, New Zealand. Cured bulbs were phenotyped for red bulb colour and freeze-dried samples were analyzed for fructan and hexose content as described elsewhere [56]. DNA was isolated from fresh leaf material or freeze-dried bulb tissue as described previously [57]. Working sets of PCR templates were generated from master stocks by whole-genome amplification using GenomiPhi V2 (GE Healthcare).

### Transcriptome sequencing

Total RNA was extracted from leaves and shoot meristem at the 4–5 leaf stage, prior to commencement of bulbing, from multiple plants of ‘CUDH2150’ and ‘Nasik Red’. Poly-A RNA was purified using Ambion Poly (A) Purist Kit (Life Technologies), as per manufacturers’ protocol.

cDNA synthesis was performed using the MINT cDNA Synthesis Kit (Evrogen). First strand synthesis was carried out on 2 μg polyA+ RNA substituting the kit 3^′^ primer with the modified primer 5^′^AAGCAGTGGTATCAACGCAGAGT(5)GT(9)CT(10)VN 3^′^. Then ds cDNA synthesis was performed with the additional 3^′^ primer 5^′^AAGCAGTGGTATCAACGCAGAGT(5)GTCT(4)GTTCTGTTTCT(4)VN at equimolar concentration to the kit “PCR Primer M1”. The optimal number of cycles was determined at 19 for Onion cDNA and 24 cycles for the kit control. After cDNA synthesis, ds cDNA was purified using the High Pure PCR Product Purification Kit (Roche). Approximately 3 μg ds cDNA was recovered from onion and 1.6 μg from the kit control. Normalization of cDNA was carried out with the Trimmer cDNA Normalization Kit (Evrogen) using 1.3 μg ds cDNA. The optimal number of cycles for the first amplification of normalized cDNA, was determined at 10 and the second amplification was performed for a total of 12 cycles. Approximately 8 μg of normalized cDNA was synthesized for sequencing. GS-FLX standard libraries were prepared from each genotype using unsheared cDNA and each was sequenced on 1/16 of a plate. Normalisation was assessed by BLASTN/X comparisons with Onion Gene Index V2.0 [58], rice and *Arabidopsis* unigene sets. A GS-FLX Titanium library was synthesized from the ‘CUDH2150’ cDNA and sequenced on a full Titanium plate. The ‘Nasik Red’ GS-FLX standard library was sequenced on full GS-FLX plate. Sequence data are accessible at NCBI under BioProject 60277. Raw flowgram data was submitted to Genbank SRA (Accession SRX031644-6).

### Bioinformatics and marker design

A reference assembly of ‘CUDH2150’ was generated by assembling adapter-trimmed reads (SRA SRX031645) using Roche Newbler V 2.0.01.14 with options -cdna -cpu 6 -minlen 45 -tr -rip -icl 100. Reads showing significant BLASTN homology (E < 10^-10^) to plant ribosomal RNA sequences were excluded from the assembly. Contigs from the assembly were filtered by length and quality using Prinseq [59] to meet the Genbank Transcriptome Shotgun Assembly (TSA) standards and submitted to TSA as accessions JR842819 – JR863573.

Polymorphisms were detected by mapping ‘Nasik Red’ reads onto the ‘CUDH2150’ reference assembly using Roche gsMapper with default parameters. Tools for parsing gsMapper 454HCDiffs.txt/454AllDiffs.txt variant output files, detecting restriction polymorphisms and performing bulk PCR primer design were developed using GNU awk, Perl and BioPython [12] and then adapted for use in the Galaxy bioinformatics framework [16-18]. These scripts along with additional helper scripts for primer analyses and format conversions are freely available for download at Github (https://github.com/cfljam/galaxy-pcr-markers/) and for installation into Galaxy at the Galaxy Toolshed (http://toolshed.g2.bx.psu.edu) as repository ‘pcr_markers’.(http://toolshed.g2.bx.psu.edu/repos/john-mccallum/pcr_markers/). Amplicon size of 90–120 bp was used for design of CAPS markers, and 60–100bp for indel and HRM markers. HRM design was limited to class I and II SNPs [60] through filtering with standard Galaxy tools.

### Marker genotyping

Initial screens of the SNP and indel markers were carried out using templates from ‘Nasik Red’, ‘CUDH2150’ and the F_1_ parent of the F_2_ population. Markers that were heterozygous in the F_1_ and segregating in an F_2_ subset of 9 lines were then tested on a core set of 93 F_2_ lines. Markers were assessed as multi-locus if multiple fragments were present after amplification with ‘CUDH2150’.

Markers were amplified by PCR using 0.5 U ThermoPrime Taq DNA polymerase (Thermo Fisher Scientific) in 15 μl reactions containing 1x PCR buffer, 200 μM dNTP, 1.5 mM MgCl_2_, 0.5 μM each primer and 20 ng template DNA. Amplifications carried out on a MasterCycler epGradientS (Eppendorf). The conditions included an initial denature at 95°C for 2 min then 40 cycles of 95°C for 30 s, 55°C for 30 s and 72°C for 30 s with a final extension of 7 min at 72°C. For CAPS markers the PCR products (5 μl) were digested in a 10 μl reaction using 3 U of restriction enzyme (NEB) (Additional file 1: Table S1) with the appropriate buffer at 1X final concentration and BSA where necessary. The digests were incubated for 3 h at 37°C or 65°C for TaqI digests. PCR and digestion products were separated using electrophoresis with a 4% agarose gel (2% Seakem LE + 2% NuSieve 3:1) and visualised under UV after ethidium bromide staining.

HRM markers were amplified in a 10 μl reaction using 1x HOT FIRE Pol EvaGreen HRM Mix (Solis BioDyne), 0.25 μM of forward and reverse primer and 20 ng DNA template. The solution was then overlaid with 15 μL PCR grade mineral oil (SIGMA). Amplification conditions included: 95°C for 15 min, then 45 cycles of 95°C for 30 s, 62°C for 30 s and 72°C for 15 s. Final hold temperatures were 95°C for 30 s and 25°C for 2 min. The products were then melted from 55°C to 95°C and melt curves assessed using the LightScanner (Idaho Technology Inc.).

SSR markers were screened and evaluated as described previously [20,28,61].

### Linkage mapping

All mapping calculations were carried out in JoinMap V4 [62] using the Kosambi function. Segregation and phase of all markers were checked and skewed markers (*p* < 0.05) were disregarded from further analysis. Linkage groups were formed using a maximum recombination fraction of 0.25 and a minimum LOD value of 7. The markers were then ordered using window size of 5 and a minimum LOD of 3. Rippling using a window size of 3 was used to visualize the marker order by both checking the minimum number of cross-overs and a maximum likelihood estimation for all possible orders. The linkage groups were then assigned a chromosome number based on the anchored SSR markers or markers that had been anchored using *A. fistulosum* - *A. cepa* monosomic addition lines [63], groups were visualized using Mapchart [64]. QTL analysis was performed using RQTL [65]. Using the framework map, a bin mapping set of 10 progeny was selected with minimization of expected bin size using the SAMPLEEXP command in MapPop [32].

### Targeted marker development and Bin mapping

Sequences for the following transcription factor families were downloaded from ‘pfam’ [66]: AP2, Dof, GRAS, HD, Myb, NAC, PHD, PLATZ, SET, Sigma70, WRKY, Whirly, BHLH, bZip, Arid and TCP. Translated assemblies of ‘CUDH2150’ transcriptome were searched for matches with these motifs using hmmsearch [67,68] with E < 10^-6^ cutoff. SNP and indel variants identified in these contigs were filtered from GFF3 formatted read mapping output using Galaxy textual filtering tools. CAPS, indel and HRM markers were designed to these using Galaxy tools described in this paper. Markers were initially tested on parental and F_1_ samples and then on a bin mapping panel of 10 individuals. Markers were assigned to genetic map bins using MapPop 1.0 [32].

## Abbreviations

CAPS: Cleaved amplified polymorphic sequence; DH: Doubled haploid; EST: Expressed sequence tag; HRM: High-resolution melting; SNP: Single nucleotide polymorphism.

## Competing interests

The authors declare that they have no competing interests.

## Authors’ contributions

SB manuscript preparation and genetic analyses. MPJ library development and molecular marker analysis. RR, KW Germplasm development, sampling, phenotyping and molecular marker analysis. ST Marker analysis, Galaxy tool development, motif searches. RC, MF Bioinformatics analyses and infrastructure. LC Galaxy tool development. RMcK Sequence analysis. JMcC Study conception and design, manuscript preparation, bioinformatics, germplasm development. All authors read and approved the final manuscript.

## Supplementary Material

Additional file 1 **Table S1.** Genetic Marker assays and map locations. Genbank accessions denote accession number of contigs or read identifier for singleton reads in SRA accession SRX031645. Click here for file

Additional file 2 **Table S2.** Bin mapped genetic marker assays targeting TF-like sequences and genomic SSR. Click here for file
